# Cantharidin attenuates the anti-β-adrenergic or anti-histaminergic or anti-serotoninergic effect of A_1_-adenosine receptor stimulation in the isolated human atrium

**DOI:** 10.1007/s00210-025-04333-2

**Published:** 2025-06-09

**Authors:** Rebecca Schwarz, Britt Hofmann, Uwe Kirchhefer, Joachim Neumann, Ulrich Gergs

**Affiliations:** 1 Institut für Pharmakologie und Toxikologie, Martin-Luther-Universität Halle-Wittenberg, Medizinische Fakultät, Magdeburger Straße 4, 06097 Halle, Germany; 2 Martin-Luther-Universität Halle-Wittenberg, Medizinische Fakultät, Ernst-Grube-Straße 40, Halle-Wittenberg, 06097 Germany; 3 Institut für Pharmakologie und Toxikologie, Medizinische Fakultät, Universität Münster, Domagkstraße, 12, 48149 Münster, Germany

**Keywords:** Cantharidin, A_1_-adenosine receptor, Human atrium, Mouse atrium, Phosphatases

## Abstract

(R)-N^6^-Phenylisopropyladenosine (R-PIA), an agonist at A_1_-adenosine receptors, per se leads to so-called “direct” negative inotropic effects (NIE) in mammalian atrium. These NIE in mammalian atrium are often accentuated in the presence of cAMP-increasing agonists acting through GTP-binding stimulatory proteins in the sarcolemma such as isoprenaline (via β-adrenoceptors), histamine (via H_2_-histamine receptors) or serotonin (via 5-HT_4_-serotonin receptors) so-called “indirect” effects. Cantharidin inhibits protein phosphatases 1 and 2A (PP1, PP2A). We hypothesized that cantharidin would attenuate this NIE of R-PIA because R-PIA activated the PPs, whereas isoprenaline, serotonin, and histamine most likely inhibit PPs. Thence, during open-heart surgery in patients suffering from severe coronary heart disease, trabeculae carneae from human right atrium (HAP) were studied. These HAPs were put into organ baths and electrically stimulated (1 Hz). For comparison, we investigated isolated electrically paced (1 Hz) left atrial preparations (LA) from wild type mice (WT) or from mice with cardiac overexpression of H_2_-histamine receptors (H_2_-TG) or mice with cardiac overexpression of the human 5-HT_4_-serotonin receptors (5-HT_4_-TG). R-PIA led to negative inotropic effects (NIE) in HAP and LA from WT in the presence of isoprenaline, which were lessened by cantharidin. Likewise, R-PIA diminished force of contraction (FOC) in HAP and in LA from H_2_-TG excited by histamine, and also these NIE were made smaller by cantharidin. Similarly, R-PIA dampened FOC in HAP and in LA from 5-HT_4_-TG aroused by serotonin, and these NIE were weakened by cantharidin. We hypothesize that A_1_-adenosine receptors shrink the positive inotropic effects of β-adrenoceptors, H_2_-histamine receptors, or 5-HT_4_-serotonin receptors, in part, by activating PP1 and/or PP2A in the human atrium.

## Introduction

**Fig. 1:**
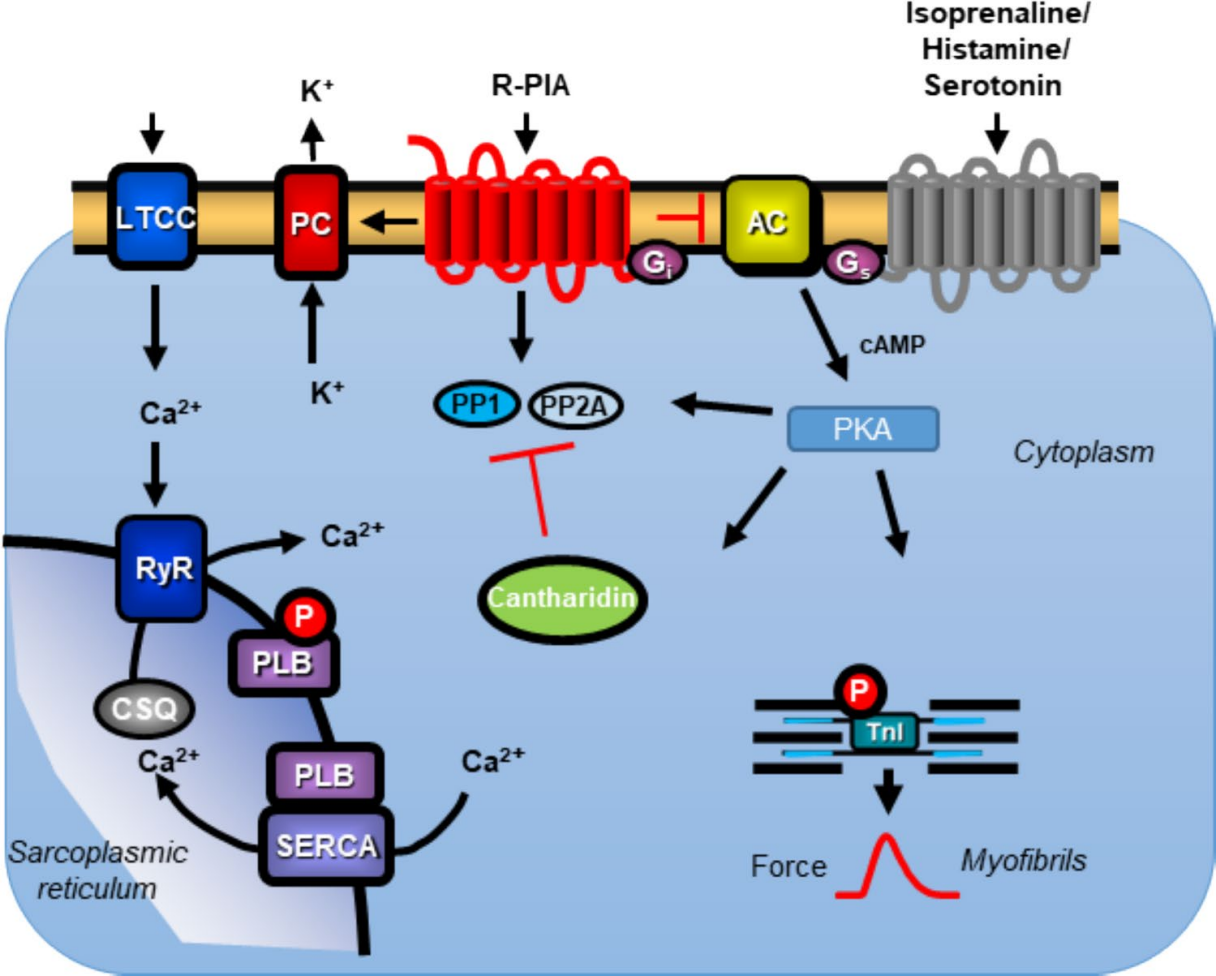
Cantharidin attenuates the negative inotropic effect of R-PIA in the presence of isoprenaline in wild type mouse left atrium.When isoprenaline stimulates β-adrenoceptors, or serotonin incites 5-HT_4_-serotonin receptors or when histamine activates H_2_-histamine receptor then via stimulatory GTP-binding proteins (Gs) and adenylyl cyclase (AC) an increase cAMP-levels in the human atrium comes about. For better oversight, we depict only a common heptahelical receptor for all three agonists. This cAMP activates cAMP-dependent protein kinases (PKA). PKA then phosphorylates regulatory proteins in the human atrium some of which inhibit protein phosphatases (PPs). The Ca^2+^ for force generation is in part derived from “trigger” Ca^2+^ through the L-type calcium cation channels (LTCC) and in larger part by subsequent release of Ca^2+^ from the sarcoplasmic reticulum (SR) via ryanodine receptors (RYR). Cardiac relaxation is brought about by phosphorylation of phospholamban (PLB) and of the inhibitory subunit of troponin. Dephosphorylations and thence inactivation of PLB and other proteins is mainly brought about by PP1 and/or PP2 A. The activities of PP1 and PP2 A are inhibited by cantharidin. R-PIA acting via A_1_-adenosine receptors may inhibit the enzymatic activity of AC via a pertussis toxin sensitive G-protein (Gi), may open potassium channels (PC), or may close the LTCC. In addition, R-PIA may directly or indirectly activate PPs

The β-adrenoceptors, H_2_-histamine receptors, and 5-HT_4_-serotonin receptors (Fig. 1) activate adenylate cyclase via stimulatory GTP-binding proteins and lead thereby to the formation of 3′,5′-cyclicadenosine monophosphate (cAMP) in the human atrium (Neumann et al. 2021, 2023). Thereafter, in the working myocardium, cAMP activates kinases (PKA) that phosphorylate and thereby activate regulatory proteins (Fig. 1). These cAMP-dependent phosphorylations of regulatory proteins like phospholamban are reversed by serine/threonine protein phosphatases (Herzig and Neumann 2000, Neumann et al. 2021).

Serine/threonine phosphatases (PP) are ubiquitously expressed in human and animal organs (Herzig and Neumann 2000). Thus, serine/threonine phosphatases are also detectable in the human atrium (Lüss et al. 2000). There might exist coupling of sarcolemmal receptors to PP. There is evidence that binding of sarcolemmal guanosine-triphosphate (GTP)-binding protein-coupled receptors to PP by scaffolding complexes may occur (Neumann et al. 2021).

We have demonstrated in guinea-pig and human cardiac preparations that cantharidin inhibited crude but also purified catalytic subunits of PP1 and PP2 A (Neumann et al. 1995a, Neumann et al.1995b, Neumann et al. 1998). Cantharidin increased force of contraction in isolated guinea-pig papillary muscle, in mouse atrial preparations, and also in human atrial and ventricular preparations via increasing the phosphorylation state of regulatory proteins like phospholamban (Neumann et al. 1995b; Linck et al. 1996; Schwarz et al. 2023).

The positive inotropic effect of isoprenaline can be attenuated in the mammalian atrium by A_1_-adenosine receptor stimulation, typically by the agonist (R)-N^6^-Phenylisopropyladenosine (R-PIA). This has been shown in the guinea-pig heart, in mouse hearts, and in human hearts (e.g., Böhm et al. 1984; Böhm et al. 1986; Böhm et al. 1994; Neumann et al. 1999; Neumann et al. 2003). This effect of R-PIA can be called anti-β-adrenergic.

However, in contrast to anti-β-adrenergic effects, to the best of our knowledge, it has not yet been reported that the PIE of serotonin or histamine in the human atrium can be attenuated by R-PIA. Therefore, this was studied here. As confirmatory experiments, we also studied how R-PIA reduced FOC that had been stimulated by isoprenaline in the wild-type mouse atrium, which we had already reported (e.g., Neumann et al. 2003).

However, in the heart of wild-type mice, histamine or serotonin do not increase the force of contraction in the mouse atrium because the H_2_ histamine receptors and the 5-HT_4_-serotonin receptors are functionally absent in the wild type mouse heart (Gergs et al. 2010, 2019). To obviate these obstacles, we had generated before mice that overexpress only in the adult heart human 5-HT_4_-serotonin receptors or human H_2_-histamine receptors. For instance, we described a positive inotropic effect (PIE) of 1 µM histamine in left atrial preparations (LA) from H_2_-TG (Gergs et al. 2019) and a PIE of 1 µM serotonin in LA from 5-HT_4_-TG (2010). Using these transgenic mice, H_2_-TG, from 5-HT_4_-TG, we could now address the question of whether R-PIA can reduce the stimulatory effect of human H_2_-histamine receptors or human 5-HT_4_-serotonin receptors in the mouse atrium here for the first time.

We have recently reported that cantharidin attenuated the negative inotropic effect of R-PIA per se (that is the absence of isoprenaline) in the isolated human atrium (Schwarz et al. 2024). Moreover, we showed that R-PIA can reduce the PIE of forskolin, a direct activator of adenylyl cyclase, or cilostamide, a phosphodiesterase inhibitor, in the human atrium (Schwarz et al. 2025). Forskolin and cilostamide elevate cAMP, but independent of an activation of GTP-binding stimulatory protein-coupled monoamine receptors (Schwarz et al. 2025). The negative inotropic effects (NIE) of R-PIA in the presence of forskolin or cilostamide were attenuated in human atria by cantharidin (Schwarz et al. 2025). The reviewers of the latter paper asked us whether this might hold true also when we used isoprenaline to elevate cAMP. We here address their interesting suggestion. We decided to use the opportunity to study other GTP-binding protein coupled receptors that increase cAMP in the human heart, like the H_2_-histamine (stimulated by histamine) and 5-HT_4_-serotonin receptors (stimulated by serotonin). In this way, we could answer the question of whether R-PIA activated phosphatases only in the absence of cAMP elevating receptor ligands (often called direct effects) or also if cAMP had been activated via GTP-binding protein-coupled receptors (often called indirect effects). We wanted to confirm any effects in the human atrium by comparing, as disease-free model systems, transgenic mouse atrial preparations.

A_1_-stimulation and M_2_-muscarinic stimulation lead often to very similar contractile effects in the isolated cardiac preparations of many mammalian species including human heart (e.g., Böhm et al. 1994). For instance, while A_1_-adenosine receptor stimulation and M_2_-muscarinic receptor stimulation both independently reduce the positive inotropic effect of isoprenaline in the mammalian heart, only M_2_-muscarinic receptor stimulated cGMP levels, but A_1_-adenosine receptor failed to increase cGMP in the mammalian heart (Böhm et al. 1984, Böhm et al. 1988, Brückner et al. 1985).

One might ask whether the present work has any clinical relevance. We would argue in an affirmatory way. It is known that in heart failure the activity of PP is enhanced and, hence, they might signal in a more effective way (Neumann et al. 1997, Klapproth et al. 2022). Moreover, currently there are experimental drugs in oncology that activate PP2 A. Such drugs should enhance the cardiac effects of A_1_-adenosine receptor stimulation. Moreover, adenosine is produced in the human heart, and the level of cardiac adenosine and its contractile effects are augmented in cardiac ischemia (Burnstock 2017).

In brief, we tested the following research questions:

Can the protein phosphatase inhibitor cantharidin attenuate the inhibitory effects of R-PIA on isoprenaline- or serotonin- or histamine-stimulated PIEs in the isolated human atrium?

## Materials and methods

### Contractile studies in mice

In brief, the right or left atrial preparations from wild-type mice or transgenic mice (5-HT_4_-TG initial description: Gergs et al. 2010, H_2_-TG: initial description: Gergs et al. 2019) were isolated and mounted in organ baths as previously described (Gergs et al. 2013; Neumann et al. 2003). All mice were aged around 180 days and were of random sex. The bathing solution of the organ baths contained 119.8 mM NaCI, 5.4 mM KCl, 1.8 mM CaCl_2_, 1.05 mM MgCl_2_, 0.42 mM NaH_2_PO_4_, 22.6 mM NaHCO_3_, 0.05 mM Na_2_EDTA, 0.28 mM ascorbic acid, and 5.05 mM glucose. The solution was continuously gassed with 95% O_2_ and 5% CO_2_ and maintained at 37 °C and pH 7.4 (Neumann et al. 1998, 2003; Kirchhefer et al. 2004). Spontaneously beating right atrial preparations from mice were used to study any chronotropic effects. The drug application was as follows. After equilibration was reached, cantharidin was added to left atrial or right atrial preparations. Then, where indicated, R-PIA was cumulatively applied to the preparations. The experiments were not done in a blinded fashion with regard to the genotype of the mice. We studied, on each experimental day, two atria. One atrium was from a transgenic animal, and the other was from a wild-type littermate mouse. In this way, we can be sure that the same reagents are used at the same hour of the day in a transgenic and a wild-type animal. Moreover, the blinding is broken when we apply histamine or serotonin. Histamine only increases the force of contraction in the left atrium from H_2_-TG and not from wild-type mice. Likewise, when we gave serotonin, only in 5-HT_4_-TG did we induce a positive inotropic effect and not in an atrium from a wild-type mouse. For better randomisation, we started the experiments on the first experimental day with a transgenic mouse and on the second experimental day with a wild type. The present mice experiments were approved by the local veterinary authorities with the permit number TM1.

## Contractile studies on human preparations

The contractile studies on human preparations were done using the same setup and buffer as used in the mouse studies (see preceding paragraph). The samples were obtained from male patients aged 62–76 years. Drug therapy included typically bisoprolol, a diuretic, apixaban, and acetylsalicylic acid. Our methods used for atrial contraction studies in human samples have been previously published and were not altered in this study (Gergs et al. 2009, 2017b, 2018; Boknik et al. 2019).

## Data analysis

Data shown are means ± standard error of the mean. Statistical significance was estimated using one-way ANOVA followed by Bonferroni’s *t*-test (for short *t*-test in the legends) as delineated in each legend. A *p*-value < 0.05 was considered to be significant. Statistical tests were performed using GraphPad Prism 9.

## Drugs and materials

The drugs cantharidin (CANT, 100 mM in dissolved dimethyl sulfoxide (DMSO)), histamine, isoprenaline, serotonin (as hydrochlorides dissolved in water), and R-PIA (dissolved in DMSO at 10 mM) were purchased from Sigma-Aldrich (Germany). In human atrial preparations, we added therefore 10 µl of the 100 mM cantharidin stock solution in a bath volume of 10 ml. Under these conditions, we reported before that we noted in some but not all human atrial muscle strips a transient negative inotropic effect which is due to DMSO, as this does not occur when we gave instead 10 µl water (Schwarz et al. 2023). After this transient NIE of DMSO, we always detected a PIE in HAP (Schwarz et al. 2023, 2025). In mouse atrial studies, we only used 3 µl of the stock solution to achieve a final concentration of 30 µM in the bath volume of 10 ml. With 3 µl, we never encountered a negative inotropic effect of DMSO in HAP (Schwarz et al. 2023). All other chemicals were of the highest purity grade commercially available. Deionized water was used throughout the experiments. Stock solutions were prepared fresh daily.

## Results

### Mouse atrium: isoprenaline

**Fig. 2:**
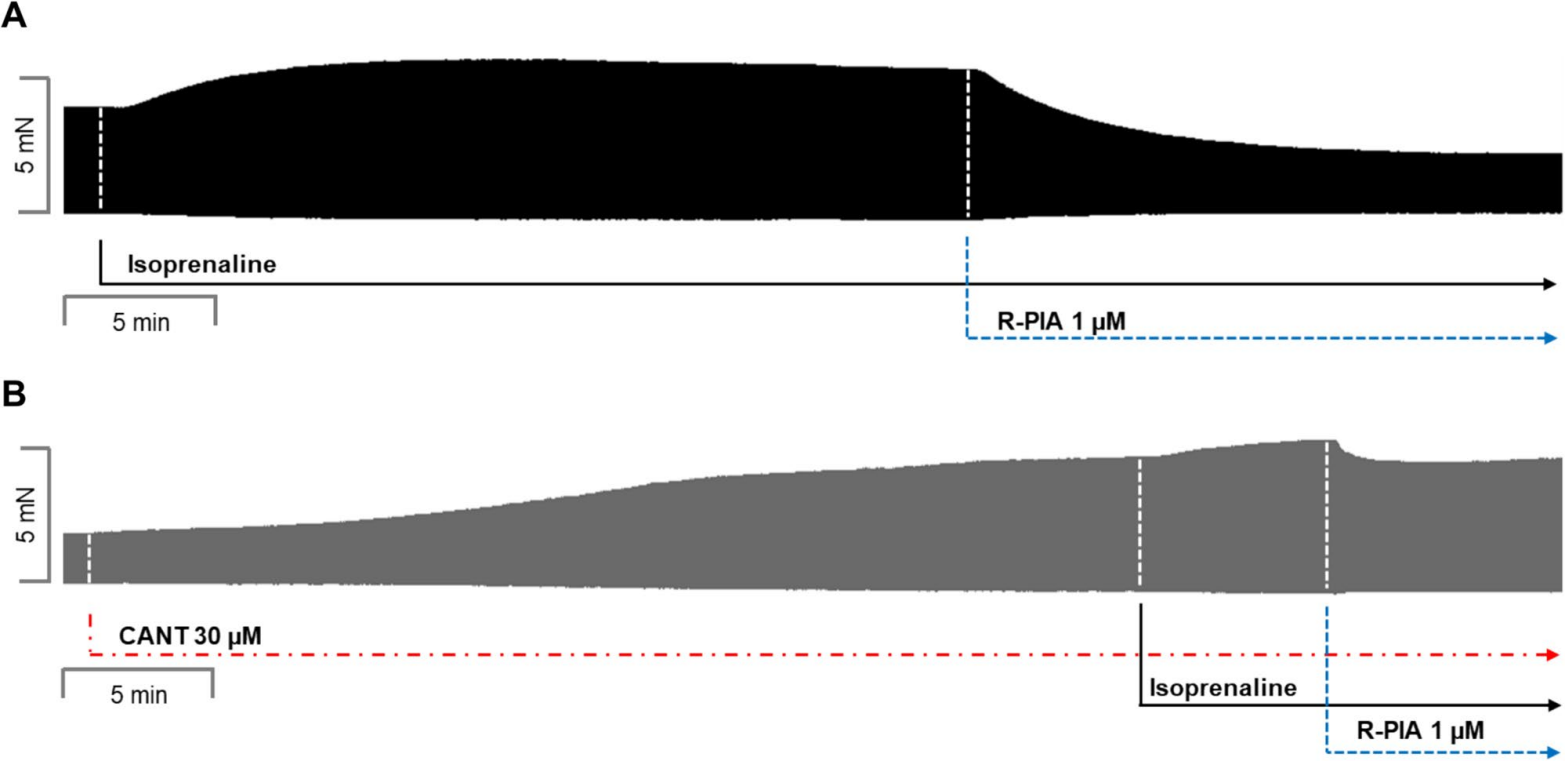
Cantharidin attenuates the negative inotropic effect of R-PIA on contractile parameters in wild type mouse left atrium. **A** Original recording of the effect of R-PIA alone on force of contraction in milli-Newton (mN, ordinate) over time in minutes (min, abscissa). First, isoprenaline (1 µM) was given and a PIE developed over time. When a plateau was reached, R-PIA was applied and thence FOC declined over time. This decline in FOC over time is quantified in Fig. 3. **B** Original recording of the effect of R-PIA on force of contraction in the presence of 30 µM cantharidin (CANT) in milli-Newton (mN, ordinate) over time in minutes (min, abscissa). First, CANT was added to the organ bath. CANT took much longer than isoprenaline (in **A**) to increase FOC. When the effect of CANT had reached a plateau with regard to FOC, then isoprenaline (1 µM) was given and a PIE developed over time. When a plateau was reached, R-PIA was applied, and thence, FOC declined over time. This decline in FOC over time is quantified in “ Fig. 3”

**Fig. 3:**
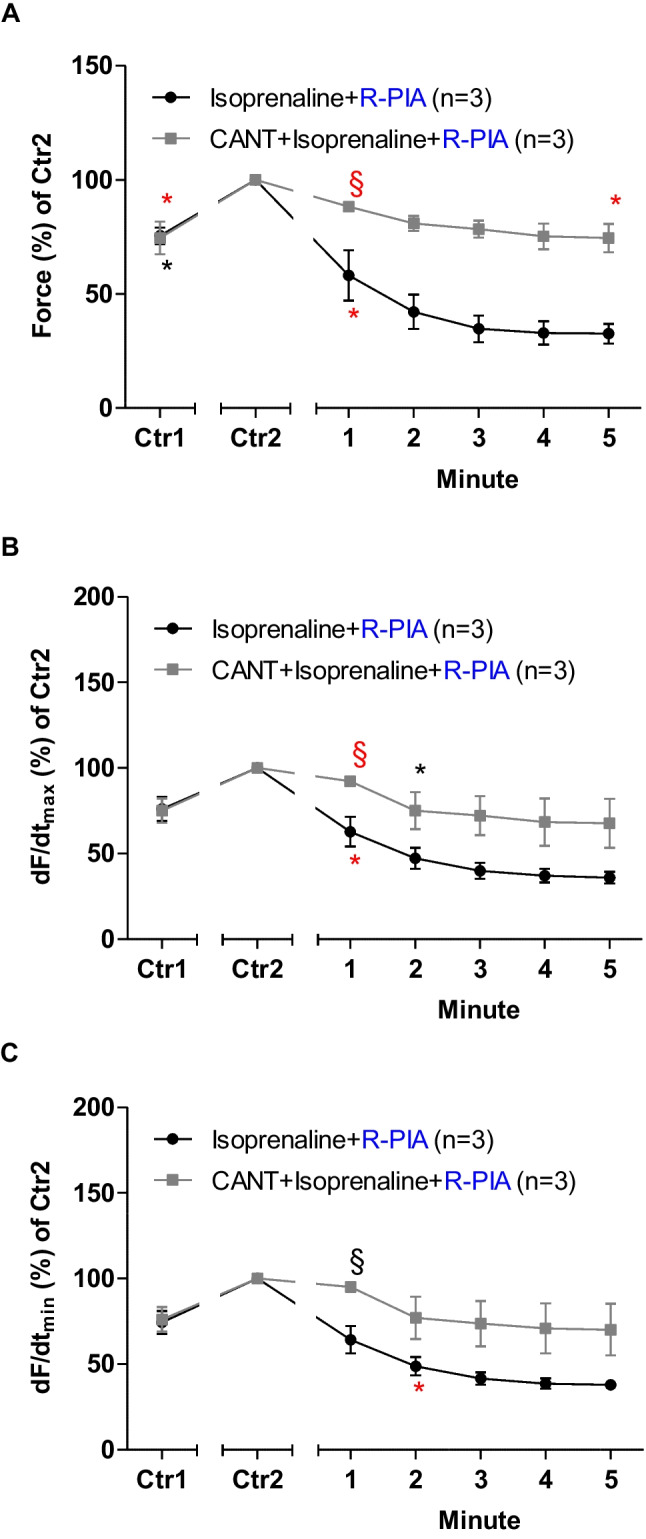
Cantharidin attenuates the negative inotropic effect of R-PIA on contractile parameters in the presence of serotonin in left atrium from H_2_-TG. **A** Quantification of the time dependence of the negative inotropic effect of R-PIA in the presence of isoprenaline alone or with the first applied cantharidin (30 µM) in isolated electrically driven mouse left atrial preparations, as depicted in Fig. 2. **B** Quantification of the time dependence effect of R-PIA on the maximum rate of tension development in the presence of isoprenaline alone or with first applied cantharidin (30 µM) in isolated electrically driven mouse left atrial preparations. **C** Quantification of the time dependence effect of R-PIA on the maximum rate of tension relaxation in the presence of isoprenaline alone or when cantharidin (30 µM) was first applied in isolated electrically driven mouse left atrial preparations. Ordinates give force of contraction (in **A**) or rate of tension development (in **B**) or rate of tension relaxation (in **C**) in percent. Abscissae indicate time after the addition of R-PIA. * and § indicate the first significant difference (*p* < 0.05) versus Ctr2 or versus force without cantharidin, respectively. Significances (*p* < 0.05) calculated with one-way ANOVA (red) and with a *t*-test (black). Number (*n*) indicates the number of experiments. Ctr1 indicates the value of pre-application of isoprenaline in the presence or absence of cantharidin. Ctr2 indicates the value of pre-application of R-PIA

R-PIA was applied, in the absence of cantharidin or after the addition of isoprenaline (original tracing: Fig. 2A) or in the presence of 30 µM cantharidin (a concentration used by us before in LA: Schwarz et al. 2023, 2024, Fig. 2B). Please note that the effect of R-PIA was time-dependent and was monophasic (Fig. 2A, B). In the presence of cantharidin, the anti-adrenergic NIE of R-PIA are attenuated (original recordings in Fig. 2A, B). These data are summarized for FOC in Fig. 3. Under these conditions, in the presence of isoprenaline (the maximum PIE of which we defined as 100%), R-PIA exerted a NIE that was larger in the absence than in the presence of cantharidin R-PIA (Fig. 3A).

When calculating the first derivative of force versus time, one noticed that the rate of tension development was enhanced by isoprenaline; then additional R-PIA was more efficient to reduce this parameter in the absence than in the presence of cantharidin (Fig. 3B). Likewise, the rate of tension relaxation was enhanced by isoprenaline, but additional R-PIA was more efficient to reduce this parameter in the absence than in the presence of cantharidin (Fig. 3C).

## Mouse atrium: histamine

**Fig. 4:**
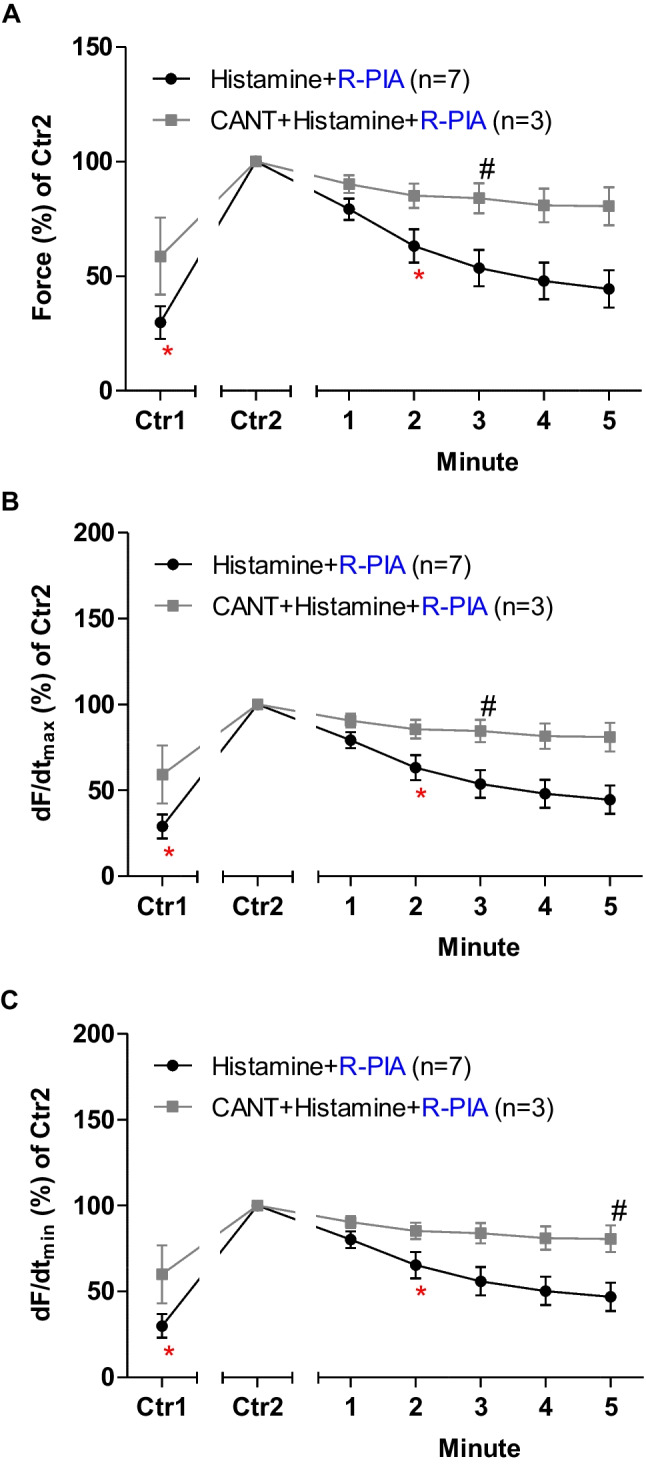
Cantharidin attenuates the negative inotropic effect of R-PIA on contractile parameters in the presence of histamine serotonin in left atrium from 5-HT_4_-TG. **A** Quantification of the time dependence negative inotropic effect of R-PIA in the presence of histamine alone or with first applied cantharidin (30 µM)in isolated electrically driven left atrium from H_2_-TG. **B** Quantification of the time dependence effect of R-PIA on the maximum rate of tension development in the presence of histamine alone or when cantharidin (30 µM) was first applied in isolated electrically driven left atrium from H_2_-TG. **C** Quantification of the time dependence effect of R-PIA on the maximum rate of tension relaxation in the presence of histamine alone or with first applied cantharidin (30 µM) in isolated electrically driven left atrium from H_2_-TG. Ordinates give force of contraction (in **A**) or rate of tension development (in **B**) or rate of tension relaxation (in **C**) in percent. Abscissae indicate time after addition of R-PIA. * and # indicate the first significant difference (*p* < 0.05) versus Ctr2 or versus without cantharidin, respectively. Significances (*p* < 0.05) were calculated with one-way ANOVA (red), with a *t*-test (black). Number (*n*) indicates the number of experiments. Ctr1 indicates value of pre-application of histamine in the presence or absence of cantharidin. Ctr2 indicates value of pre-application of R-PIA. * and + indicate the first significant difference versus 30 µM cantharidin or time-matched control values (Ctr2) or R-PIA in presence of cantharidin, respectively. The number in brackets indicates the number of experiments. Ctr1 indicates pre-drug value. Ctr2 (100%) indicates maximum effects to serotonin in the presence of absence of cantharidin

Next, R-PIA was again applied, but now in the absence of cantharidin and after the addition of histamine in LA from H_2_-TG (Fig. 4). In the presence of cantharidin, the anti-histaminergic NIE of R-PIA were attenuated (Fig. 4). Under these conditions, in the presence of histamine, R-PIA exerted NIE that were larger in the absence than in the presence of cantharidin R-PIA (Fig. 4A).

When we look at the first derivative of force versus time, we notice that the rate of tension development was amplified by cantharidin and isoprenaline, but additional R-PIA was more efficient to make smaller this parameter in the absence than in the presence of cantharidin (Fig. 4B). Likewise, the rate of tension relaxation was enhanced by cantharidin in the presence of isoprenaline, but additional R-PIA was more efficient to attenuate this parameter in the absence than in the presence of cantharidin (Fig. 4C).

## Mouse atrium: serotonin

**Fig. 5:**
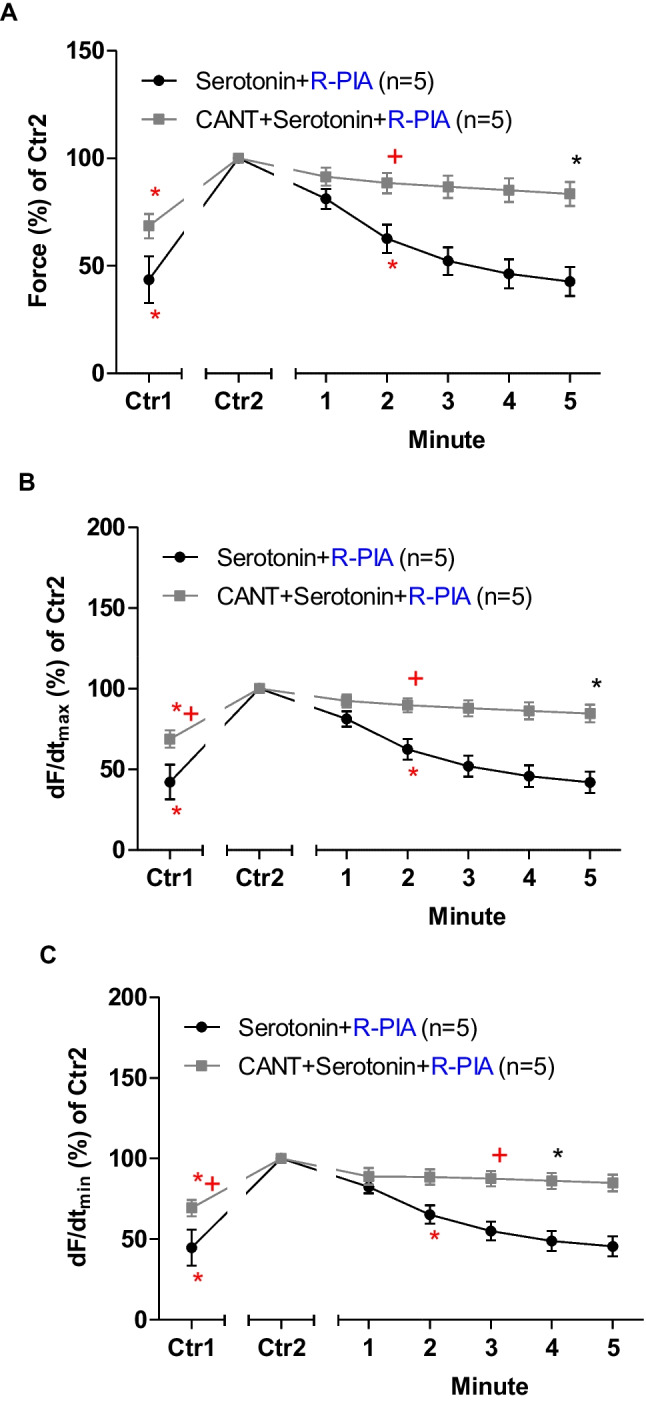
Cantharidin attenuates the negative inotropic effect of R-PIA on contractile parameters in the presence of isoprenaline in human atrium. **A** Quantification of the time dependence negative inotropic effect of R-PIA in the presence of serotonin alone or when cantharidin (30 µM) was first applied in isolated electrically driven left atrium from 5-HT_4_-TG. **B** Quantification of the time dependence effect of R-PIA on the maximum rate of tension development in the presence of serotonin alone or with first applied cantharidin (30 µM) in isolated electrically driven left atrium from 5-HT_4_-TG. **C** Quantification of the time dependence effect of R-PIA on the maximum rate of tension relaxation in the presence of serotonin alone or when cantharidin (30 µM) was first applied in isolated electrically driven left atrium from 5-HT_4_-TG. Ordinates give force of contraction (in **A**) or rate of tension development (in **B**) or rate of tension relaxation (in **C**) in percent. Abscissae indicate time after addition of R-PIA. * and + indicate the first significant difference (*p* < 0.05) versus Ctr2 or versus without cantharidin, respectively. Significances (*p* < 0.05) were calculated with one-way ANOVA (red), with a t-test (black). Number (*n*) indicates number of experiments. Ctr1 indicates value of pre-application of serotonin in the presence or absence of cantharidin. Ctr2 indicates value of pre-application of R-PIA

In the next set of experiments, R-PIA was applied, after the addition of serotonin in LA from 5-HT_4_-TG in the absence or in the presence of cantharidin (Fig. 5). In the presence of cantharidin, the anti-serotoninergic NIEs of R-PIA were attenuated (Fig. 5A). When calculating the first derivative of force versus time, one noticed that the rate of tension development was enhanced by cantharidin and isoprenaline, but additional R-PIA was more efficient to diminish this parameter in the absence than in the presence of cantharidin (Fig. 5B). Likewise, the rate of tension relaxation was enhanced by cantharidin in the presence of isoprenaline, but additional R-PIA was more efficient to lessen this parameter in the absence than in the presence of cantharidin (Fig. 5C).

## Human right atrial preparations: isoprenaline

**Fig. 6:**
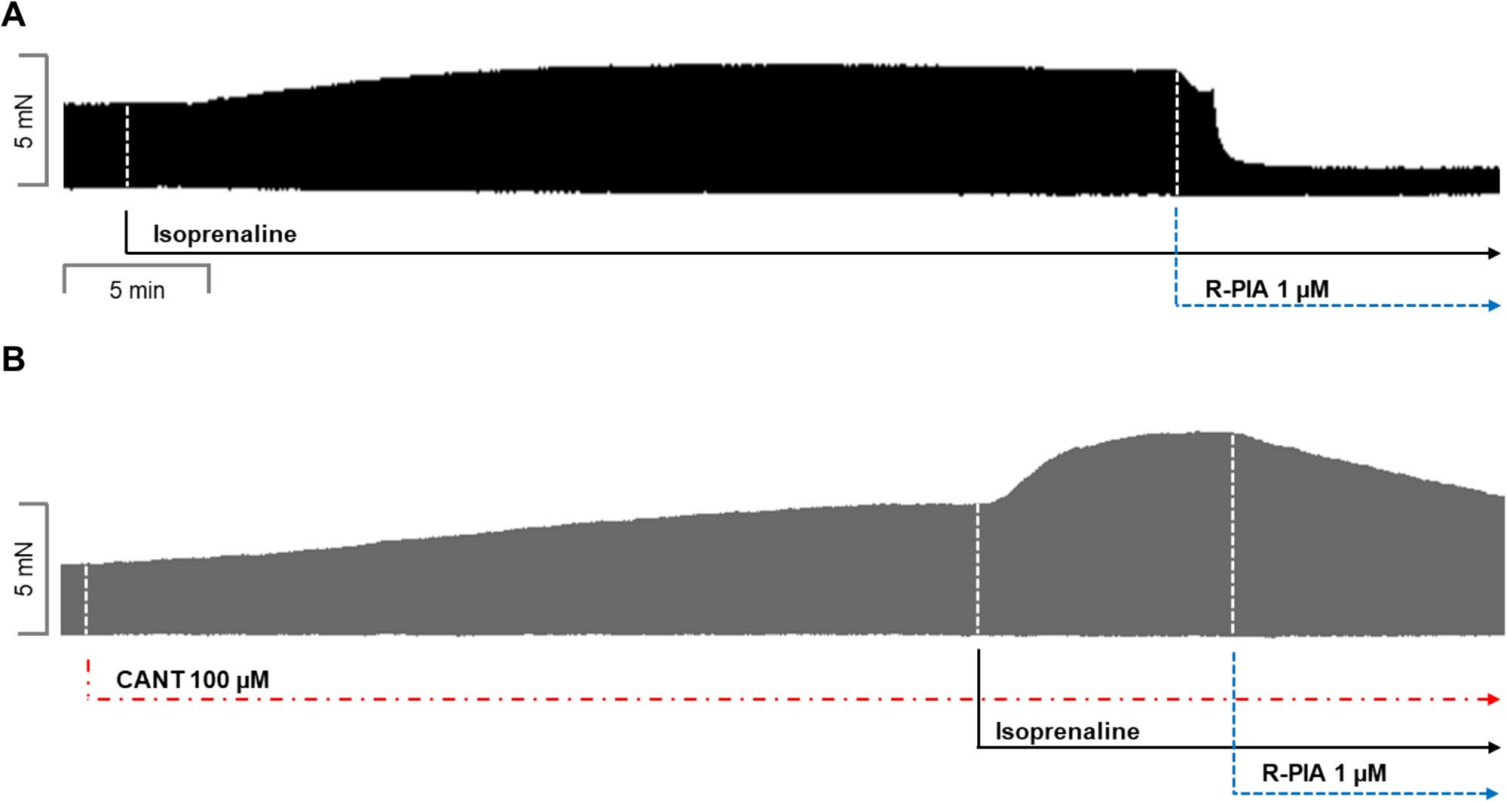
Cantharidin attenuates the negative inotropic effect of R-PIA on contractile parameters in the presence of isoprenaline in human atrium. **A** Original recording of the effect of R-PIA alone in the presence of isoprenaline on force of contraction in milli-Newton (mN, ordinate) over time in minutes (min, abscissa). **B** Original recording of the effect of R-PIA in the presence of isoprenaline on force of contraction in the presence of 100 µM cantharidin in milli-Newtons (mN, ordinate) over time in minutes (min, abscissa).

**Fig. 7:**
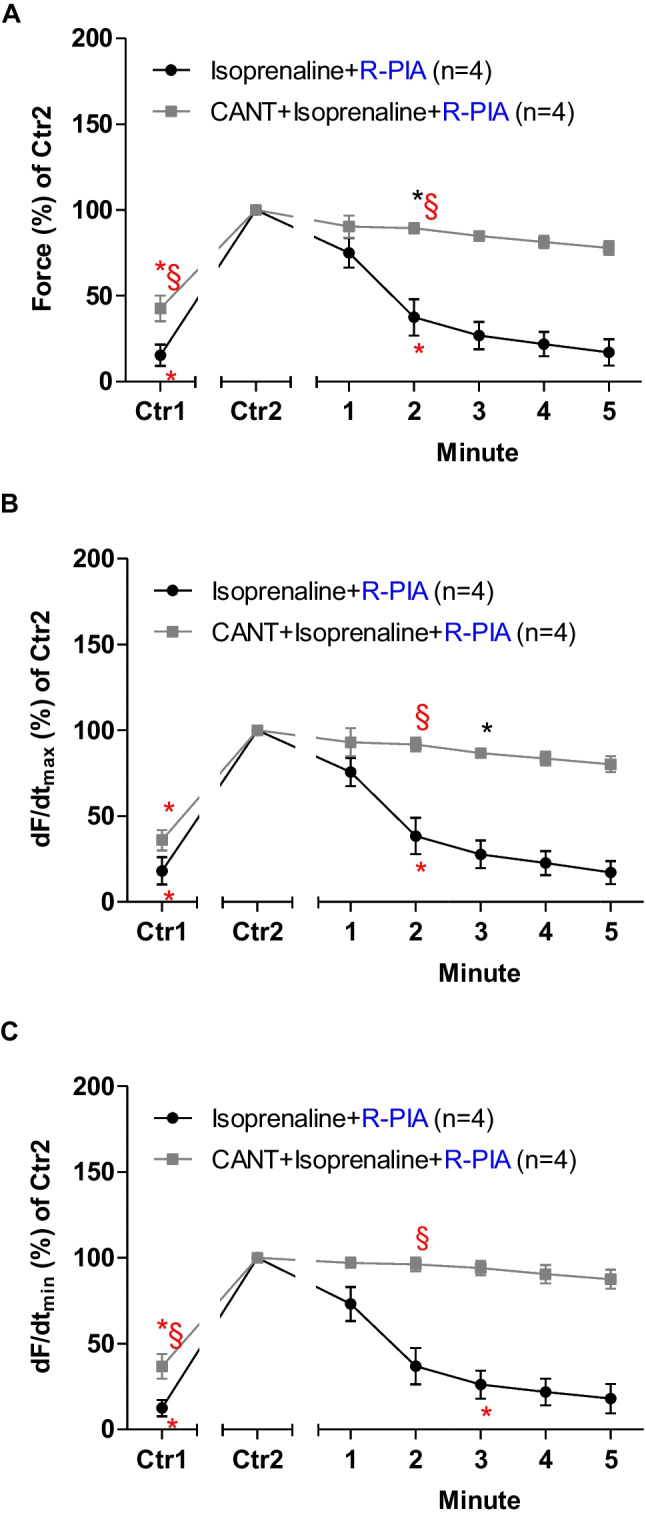
Cantharidin attenuates the negative inotropic effect of R-PIA on contractile parameters in the presence of histamine in human atrium. **A** Quantification of the time dependence negative inotropic effect of R-PIA in the presence of isoprenaline alone or with first applied cantharidin (100 µM) in isolated electrically driven human atrial preparations, as depicted in Fig. 3. **B** Quantification of the time dependence effect of R-PIA on the maximum rate of tension development in the presence of isoprenaline alone or when cantharidin (100 µM) was first applied in isolated electrically driven human atrial preparations. **C** Quantification of the time dependence effect of R-PIA on the maximum rate of tension relaxation in the presence of isoprenaline alone or with first applied cantharidin (100 µM) in isolated electrically driven human atrial preparations. Ordinates give force of contraction (in **A**) or rate of tension development (in **B**) or rate of tension relaxation (in **C**) in percent. Abscissae indicate time after addition of R-PIA. * and § indicate the first significant difference (*p* < 0.05) versus Ctr2 or versus without cantharidin, respectively. Significances calculated with one-way ANOVA (red), with a *a*-test (black). Number (*n*) indicates the number of experiments. Ctr1 indicates value of pre-application of isoprenaline in the presence or absence of cantharidin. Ctr2 indicates value of pre-application of R-PIA

The same protocol was used in human atrial preparations as in mice (vide supra). Again, a single concentration of cantharidin, but 100 µM, based on our previous studies (Schwarz et al. 2024) was utilized. Thereafter, isoprenaline raised FOC; then R-PIA diminished FOC. Like in mouse atrium, the effect of R-PIA is monophasic (Fig. 6). However, in the presence of cantharidin, the negative inotropic effect of R-PIA in the presence of isoprenaline is attenuated (Fig. 6A versus Fig. 6B). These data are summarized for the NIE in the presence of isoprenaline (Fig. 7A). In the presence of isoprenaline, the reductions by R-PIA of the rate of tension development and the time of relaxation were attenuated by cantharidin (Fig. 7B, C).

## Human right atrial preparations: histamine

**Fig. 8:**
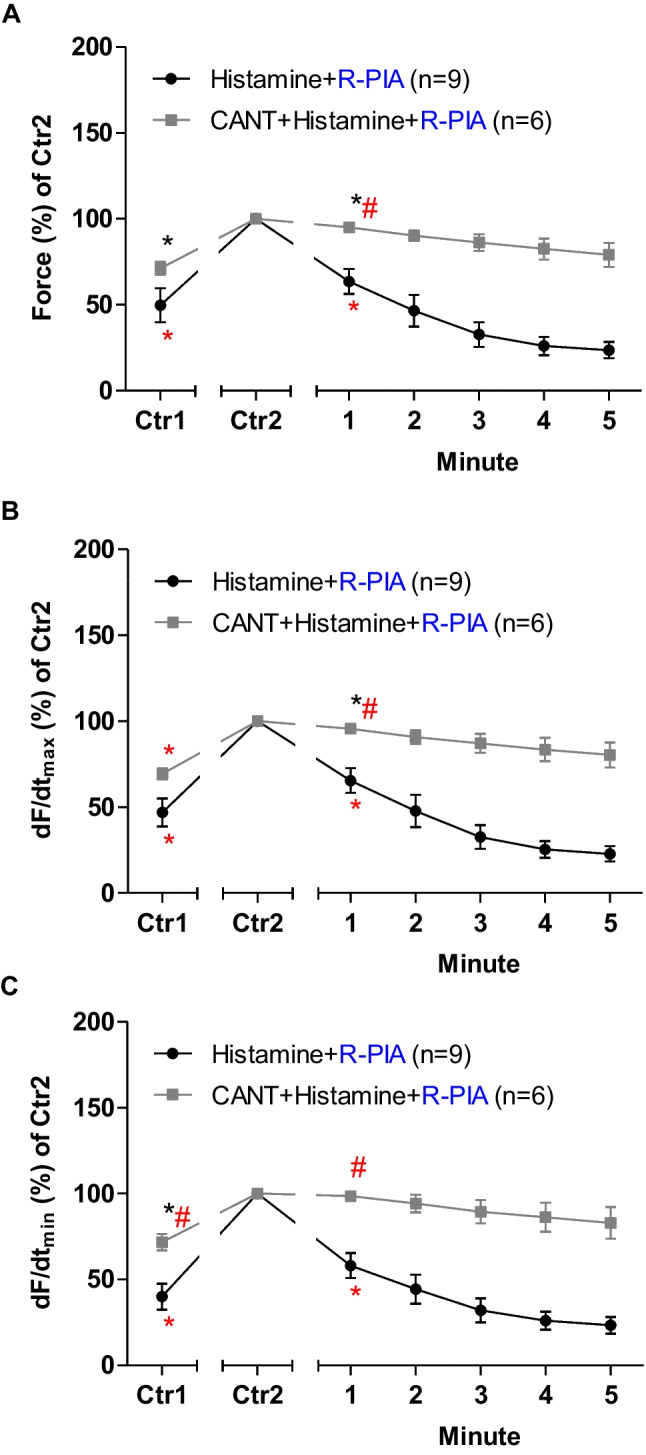
Cantharidin attenuates the negative inotropic effect of serotonin on contractile parameters in the presence of serotonin in human atrium. **A** Quantification of the time dependence negative inotropic effect of R-PIA in the presence of histamine alone or when cantharidin (100 µM) was first applied in isolated electrically driven human atrial preparations. **B** Quantification of the time dependence effect of R-PIA on the maximum rate of tension development in the presence of histamine alone or with first applied cantharidin (100 µM) in isolated electrically driven human atrium. **C** Quantification of the time dependence effect of R-PIA on the maximum rate of tension relaxation in the presence of histamine alone or when cantharidin (100 µM) was first applied in isolated electrically driven human atrial preparations. Ordinates give force of contraction (in **A**) or rate of tension development (in **B**) or rate of tension relaxation (in **C**) in percent. Abscissae indicate time after addition of R-PIA. * and # indicate the first significant difference (*p* < 0.05) versus Ctr2 or versus without cantharidin, respectively. Significances (*p* < 0.05) were calculated with One-Way ANOVA (red), with a *t*-test (black). Number (*n*) indicates number of experiments. Ctr1 indicates value of pre-application of histamine in presence or absence of cantharidin. Ctr2 indicates value of pre-application of R-PIA

In a similar fashion as in LA from H_2_-TG, histamine was given and then R-PIA was applied; R-PIA elicited a pronounced negative inotropic effect (Fig. 8). However, in the presence of cantharidin and in the presence of histamine, the negative inotropic effect of R-PIA is attenuated (Fig. 8). These data are summarized for the negative inotropic effects in the presence of histamine (Fig. 8). In the presence of histamine, the reductions by R-PIA of the rate of tension development and the time of relaxation were attenuated by cantharidin (Fig. 8A, B).

## Human right atrial preparations: serotonin

**Fig. 9:**
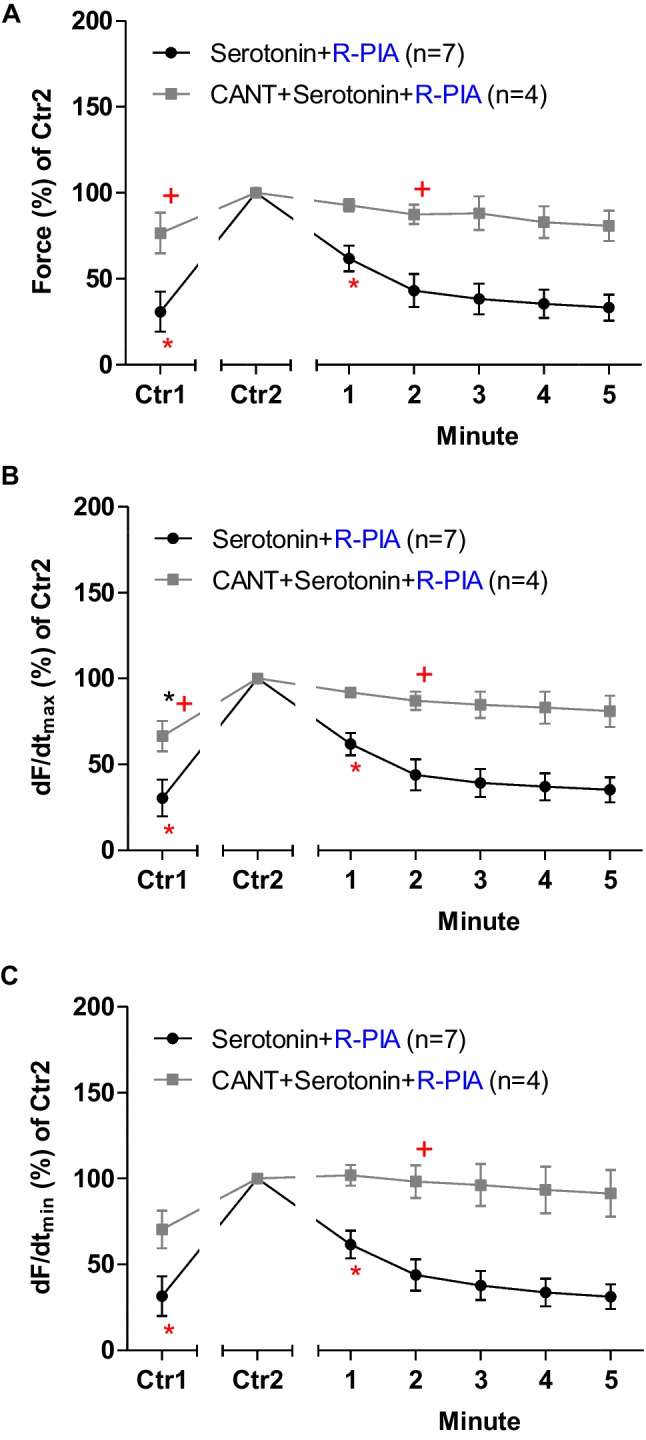
**A** Quantification of the time dependence negative inotropic effect of R-PIA in the presence of serotonin alone or with first applied cantharidin (100 µM) in isolated electrically driven human atrial preparations. **B** Quantification of the time dependence effect of R-PIA on the maximum rate of tension development in the presence of serotonin alone or when cantharidin (100 µM) was first applied in isolated electrically driven human atrial preparations. **C** Quantification of the time dependence effect of R-PIA on the maximum rate of tension relaxation in the presence of serotonin alone or with first applied cantharidin (100 µM) in isolated electrically driven human atrium. Ordinates give force of contraction (in **A**) or rate of tension development (in **B**) or rate of tension relaxation (in **C**) in percent. Abscissae indicate time after addition of R-PIA. * and + indicate the first significant difference (*p* < 0.05) versus Ctr2 or versus without cantharidin, respectively. Significances were calculated with one-way ANOVA (red), with a *t*-test (black). Number (*n*) indicates number of experiments. Ctr1 indicates value of pre-application of serotonin in presence or absence of cantharidin. Ctr2 indicates value of pre-application of R-PIA

Finally, as in 5-HT_4_-TG, serotonin was given and then R-PIA was applied. R-PIA elicited a pronounced negative inotropic effect in the presence of serotonin (Fig. 9). However, in the presence of cantharidin and in the presence of serotonin, the NIE of R-PIA are lessened. These data are summarized for the NIE in the presence of serotonin (Fig. 9A). Under these conditions, cantharidin like serotonin boosted the rate of tension development and the rate of relaxation: In the presence of serotonin, the diminishments by R-PIA of the rate of tension development and the time of relaxation were eased by cantharidin (Fig. 9B, C).

## Discussion

The main new findings in this report are as follows: cantharidin attenuates the anti-β-adrenergic, the anti-serotoninergic, or the anti-histaminergic negative inotropic effects of R-PIA in the human atrium. The positive inotropic effects of isoprenaline, histamine, and serotonin in the human atrium differ and are brought about by different receptors in the sarcolemma (Fig. 1). These positive inotropic effects have in common that they are due to an activation of adenylyl cyclase via stimulatory GTP-binding protein after receptor occupation and lead to increased production of cAMP. This cAMP then leads via cAMP-dependent protein kinase to phosphorylation and an activation of cardiac regulatory proteins (Fig. 1). Such phosphorylations are reversed by PP. In most mammals, such as mice and men, the PP1 and PP2 A are responsible for 90% reversal of protein phosphorylations at the amino acids serine or threonine (Herzig and Neumann 2000). Other PPs like PP2B, PP2 C, and PP5 play an additional, but minor role in the human heart that is currently not well defined and was not studied here (Neumann et al. 2021).

## Role of isoprenaline

In a mouse atrium and human atrium, the anti-β-adrenergic inotropic effects of R-PIA were attenuated by previously applied cantharidin. There is evidence that isoprenaline reduced the activity of cardiac PP1 by increasing the phosphorylation state of phosphatase inhibitor 1 (Ahmad et al. 1989, Neumann et al. 1991). The observation that the anti-β-adrenergic effects of R-PIA were attenuated by previously applied cantharidin in HAP is not a proof that the action of cantharidin is solely due to inhibition of phosphatases, but it is, in our eyes, a reasonable working hypothesis that requires further biochemical studies. It may be kept in mind that there are regional differences in the anti-β-adrenergic role of A_1_-adenosine receptors in the mammalian heart: in guinea pig and human ventricular preparations, in contrast to atrial preparations from these two species, R-PIA alone does not decrease the force of contraction (Burnstock 2017). However, when the force was pre-stimulated by isoprenaline, R-PIA induced a time- and concentration-dependent negative inotropic effect in guinea pig papillary muscle or HAP (e.g., Neumann et al. 1995a, Böhm et al. 1994). Hence, our findings for the anti-β-adrenergic effects of R-PIA in atrial preparations from mice and man may not apply to the mammalian ventricle, but we strongly suspect that we studied here a general mechanism active also in the human ventricle because all relevant enzymes (PPs, cAMP dependent protein kinase, phosphatase inhibitor 1) are also found in the human ventricle and not only in the human atrium, but possibly to a different extent.

## Role of serotonin

While it is well established that adenosine and R-PIA reduce the positive inotropic effects of isoprenaline in the animal and human heart (vide supra), no research on a cardiac interaction of the effects of serotonin and the A_1_-adenosine receptor agonist R-PIA is available, to the best of our knowledge. This may stem from the fact that 5-HT_4_-serotonin receptors are only functional in porcine, monkey, and human atria and not in typical experimental animals such as guinea pigs or in rat atria or wild-type mouse atria (Neumann et al. 2023). However, since the seminal paper of Kaumann’s group (Kaumann et al. 1990), it is well accepted that serotonin increases FOC in the HAP only via 5-HT_4_-serotonin receptors (Kaumann and Levy 2006). These stimulations of 5-HT_4_ receptors then lead to an increase in cAMP and an elevation of the phosphorylation state of, e.g., phospholamban (Fig. 1) in HAP (Gergs et al. 2009). Furthermore, it is well known that phosphorylated phospholamban is dephosphorylated by PP1 and PP2 A (Herzig and Neumann 2000). Hence, it seems that R-PIA reduces the force of contraction raised by serotonin in HAP, but it is reported here for the first time. In subsequent studies, one should study whether this reduction in FOC in HAP is accompanied (and thus possibly caused) by a reduction in the serotonin-stimulated phospholamban phosphorylation by R-PIA. As mentioned in the introduction, A_1_-adenosine receptors and M_2_-muscarinic receptors share many similarities in their contractile effects in the human atrium. In this context, one might consider that, indeed, muscarinic stimulation is well known to antagonize the positive contractile effect of serotonin even in electrically stimulated isolated human atrial cardiomyocytes (Sanders et al. 1995). Hence, the NIE of R-PIA and carbachol after serotonin stimulation may indicate similar signal transduction pathways in HAP that should be elucidated in subsequent studies.

## Role of histamine

It has been shown that the positive inotropic effect of histamine acting via H_2_-histamine receptors in guinea pig isolated hearts or human atrial preparations could be antagonized by adenosine (Baumann et al. 1981, Genovese et al. 1988). Moreover, adenosine could inhibit the histamine-stimulated adenylyl cyclase activity in broken cell preparations of the guinea pig ventricle (Baumann et al. 1981) and the histamine-stimulated current through L-type calcium cation currents in guinea-pig ventricular cardiomyocytes (Belevych et al. 2001). We have presented evidence that histamine can phosphorylate and thus activate a protein called inhibitor 1 in the ventricle of H_2_-TG (Gergs et al. 2019). Phosphorylated inhibitor 1 is known to inhibit the enzymatic activity of protein phosphatase 1 (Ahmad et al. 1989, Neumann et al. 1991). Thus, these data suggest that H_2_-histamine receptors inactivate cardiac PP1 (Gergs et al. 2019). It is likely (because cAMP protein kinase is activated by 5-HT_4_-serotonin receptors: Kaumann and Levy 2006), but as far as we know, it was not published that serotonin in the mammalian heart can also inhibit PP1 and possibly also PP2 A via the same mechanisms used for isoprenaline (Ahmad et al. 1989, Neumann et al. 1991).

## Role of adenosine or R-PIA

The cardiac action of R-PIA is different between species and between cardiac regions, namely atrium and ventricle. If we look firstly at the atrium, functionally negative inotropic effects of R-PIA in the absence and in the presence of isoprenaline are well established in human atrium and mouse atrium (e.g., Böhm et al. 1994, Boknik et al. 2009, Du et al. 1994, Neumann et al. 2003). The negative inotropic effects of R-PIA vanish in A_1_-adenosine receptor-knockout mice and thus are A_1_-adenosine receptor mediated (review: Burnstock 2017). Similar findings are seen in human atrial samples; here, studies with A_1_-adenosine receptor specific antagonists come to the same conclusions (Böhm et al. 1989). Thus, on the one hand, our anti-adrenergic negative inotropic effects of R-PIA in mouse atrium and human atrium are probably A_1_-adenosine receptor mediated. On the other hand, we have repeatedly shown that the negative inotropic effect of R-PIA in the presence of isoprenaline in the guinea pig ventricle involves phosphorylation of the phosphatase inhibitor 1 and subsequent inhibition of PP1 (Gupta et al. 1993, 1994, 1998, Neumann et al. 1994). Others presented evidence that in the rat ventricle, R-PIA can also activate PP2 A (Liu and Hofmann 2002, Tikh et al. 2008). Hence, there is precedence that R-PIA can activate PP1 and/or PP2 A that was previously inhibited by isoprenaline in the mammalian heart. We postulate now that the same occurs in the human atrium. Isoprenaline, serotonin, and histamine may inhibit PP1 or/and PP2 A in the human atrium and R-PIA tries to re-activate PP1 and PP2 A in the human atrium, but this re-activation is impaired by the addition of cantharidin.

## Role of transgenic mice

One might ask why we used transgenic mice in addition to human atrium. We regard these experiments as confirmatory. In wild-type mice, isoprenaline is active to raise force of contraction, but histamine or serotonin cannot increase atrial FOC (Gergs et al. 2010, Gergs et al. 2019). Hence, on the one hand, in wild-type mice, one can study the function of the β-adrenoceptor on PP without interference from H_2_- or 5-HT_4_-receptors. On the other hand, H_2_-TG offer the chance to study histamine effects without interference from serotonin receptors. Of course, 5-HT_4_-TG offer the opportunity to investigate serotonin without interference from histamine receptors. Moreover, these mice have no visible cardiac defects or comorbidities and are not pre-treated by drugs, in contrast to our HAP from diseased patients. We had shown before that R-PIA attenuates the effect of isoprenaline on FOC in wild type mice (Neumann et al. 1999, 2003). We also reported that the effects of R-PIA are more potent in mice with cardiac overexpression of A_1_-adenosine receptors (Neumann et al. 2003). However, to the best of our knowledge, we offer here the first evidence that the PIE of histamine in H_2_-TG is antagonized by R-PIA. Histamine and serotonin have in common that they are also to some extent produced like noradrenaline in the human heart. Histamine exerts a PIE via H_1_- and H_2_-histamine receptors in the human atrium (Gergs et al. 2017, Rayo et al. 2024). However, only H_2_- but not H_1_-receptor stimulation leads to a cAMP increase in the mammalian heart and more specifically to phospholamban phosphorylation in the human atrium (Gergs et al. 2019, Rayo Abella et al. 2024). Therefore, we decided to study the H_2_-histamine receptor here. Similarly, in the human atrium, the PIE of serotonin is solely mediated by 5-HT4 receptors (Kaumann and Levy 2006), and therefore, we used 5-HT_4_-TG to study the role of serotonin in the present work.

Comparing this present study with a past study where we elevated cAMP with forskolin and cilostamide, we conclude that irrespective of how cAMP is increased, this cAMP probably inhibited PPs and then PPs are re-activated by A_1_-adenosine receptor stimulation. For comparison, we plan to study in a similar manner the M_2_-receptor stimulation in HAP, which often signals similarly to A_1_-adenosine receptor stimulation in the human atrium, and this might tell us whether we detected a common theme between these receptors.

### Limitations of the study

We cannot offer a clear biochemical chain of events from the receptors to the PPs in the human atrium. We do not know whether cantharidin acts in a similar way in the human ventricle as in the human atrium, because we currently have no access to human ventricular tissue.

In summary, we can now answer the hypotheses put forward in the Introduction in the following way. Cantharidin attenuates the NIE of R-PIA (an A_1_-adenosine receptor agonist) after stimulation of β-adrenoceptors or H_2_-histamine receptors or 5-HT_4_-receptors in the (transgenic) mouse and human atrium. We suggest that after cAMP-elevating G-protein coupled receptors had inhibited PPs in HAP, subsequently applied PIA activated PPs. Cantharidin would inhibit cardiac PPs in HAP that would otherwise be stimulated by A_1_-adenosine receptor.

## Data Availability

Availability of data and materials: The data of this study are available from the corresponding author upon reasonable request.
